# Mechanism of BaP- regulated hepatic lipid metabolism via AhR activation and AR modulation

**DOI:** 10.3389/fphar.2026.1880959

**Published:** 2026-09-21

**Authors:** Han Wang, Mengdi Zhang, Xue Wang, Min Su, Lei Fan, Yulu Du, Yuxia Hu, Xiaoli Lv, Jun Li, Shuhong Zhou, Baoquan Bao, Tuya Bai, Weizhong Huangfu, Fuhou Chang

**Affiliations:** 1 School of Pharmacy, Inner Mongolia Medical University, Hohhot, China; 2 Inner Mongolia Autonomous Region Engineering Research Center for New Drug Screening, Inner Mongolia Medical University, Hohhot, China; 3 The Affiliated Hospital of Inner Mongolia Medical University, Hohhot, China; 4 Pharmacy Teaching-Research Section, School of Medicine, Qingdao Binhai University, Qingdao, China

**Keywords:** benzo[a]pyrene, aryl hydrocarbon receptor, androgen receptor, androgens, lipid metabolism

## Abstract

**Objective:**

Benzo[a]pyrene (BaP) is a ubiquitous environmental pollutant that disturbs hepatic lipid metabolism. The androgen receptor (AR) protects hepatic lipid homeostasis, but whether BaP interferes with AR nuclear translocation through aryl hydrocarbon receptor (AhR) activation to cause metabolic disorders remains unknown *in vivo* and *in vitro*. This study aimed to clarify BaP-mediated regulation of AR signaling and its dependence on the AhR pathway during hepatic lipid metabolism.

**Methods:**

An androgen-deficient mouse model was established by bilateral orchiectomy, and lipid disorders were induced by BaP (0.9 mg kg^−1^ day^−1^). HepG2 cells were treated with BaP (1 μmol L^−1^) with or without dihydrotestosterone (DHT, 1 μmol L^−1^) or AhR antagonist CH223191 (1 μmol L^−1^). Hepatic injury was evaluated by HE and Oil Red O staining. Serum lipids, liver function, AR nuclear translocation, and expression of lipid-related genes were measured.

**Results:**

In androgen-present mice, BaP induced liver injury and lipid accumulation, inhibited AR nuclear translocation, reduced β-oxidation genes (PGC-1α, CPT1A, PPARα), and increased lipogenic genes (SREBP2, HMGCR, LXRα). In androgen-deficient mice, BaP promoted AR translocation and improved lipid metabolism. In HepG2 cells, BaP alone enhanced AR translocation and fatty acid oxidation, while BaP + DHT inhibited AR translocation and promoted lipogenesis. All effects were reversed by CH223191.

**Conclusion:**

BaP exerts bidirectional regulation on AR via AhR: it exerts an anti-androgenic in androgen-replete conditions and regulates AR-related signaling pathways in androgen deficiency. The AhR–AR axis is a key target for preventing BaP-induced hepatic steatosis.

## 1 Introduction

Disorders of lipid metabolism are a major contributing factor to a wide range of metabolic diseases, including metabolic dysfunction–associated steatotic liver disease (MASLD). MASLD encompasses a pathological spectrum ranging from simple hepatic lipid accumulation to hepatic steatosis, metabolic dysfunction–associated steatohepatitis, cirrhosis, and ultimately hepatocellular carcinoma (HCC) (Zhou and Sun, 2021; Zhu et al., 2023; Powell et al., 2021). A recent meta-analysis reported that the global prevalence of MASLD is approximately 37%, with a rapidly increasing incidence and a higher prevalence among males (Riazi et al., 2022). Multiple factors contribute to dysregulated hepatic lipid synthesis and metabolism, among which unhealthy lifestyles and environmental pollution have been recognized as major aggravating factors for MASLD progression (Zhu et al., 2025). Epidemiological studies indicate that cigarette smoking is a significant risk factor for hepatic lipid metabolic disorders (Argenziano et al., 2024), as cigarette smoke can disrupt hepatic lipid homeostasis and promote triglyceride accumulation in the liver (Zhang J. et al., 2025).

BaP, a major toxic constituent of cigarette smoke, is a representative environmental pollutant and has been implicated as a trigger for tumorigenesis, inflammation, and metabolic diseases (Bukowska and Sicińska, 2021). Increasing evidence suggests that environmental contaminants such as BaP and 2,3,7,8-tetrachlorodibenzo-p-dioxin (TCDD) are closely associated with the development of MASLD (Post et al., 2025; Bu et al., 2024; Zhang M. et al., 2025). BaP binding to the AhR has been shown to induce spontaneous hepatic steatosis in mice (Treviño and Katz, 2018). As a classical AhR ligand, BaP activates AhR, which subsequently heterodimerizes with the AhR nuclear translocator (ARNT), translocates into the nucleus, and induces the transcription of downstream target genes, including cytochrome P450 1A1 (CYP1A1) (Wang et al., 2021). Our previous studies demonstrated that BaP may promote hepatic lipid synthesis by upregulating genes involved in lipogenesis and lipid transport through AhR activation in liver tissue (Lou et al., 2022).

AhR mediates a broad range of toxicological effects, including endocrine disruption and carcinogenesis (Zo et al., 2024; Zuberi et al., 2023; Xu et al., 2024; Wang et al., 2025), while also participating in diverse physiological processes such as lipid metabolism, cell cycle regulation, and immune function (Zheng et al., 2025). Notably, AhR shares regulatory cofactors with steroid hormone receptors and can interact with estrogen receptors, AR, and glucocorticoid receptors, thereby modulating their biological functions (Ohtake et al., 2007). Environmental toxicants can exploit the E3 ubiquitin ligase activity of AhR to exert dual regulation over target proteins by promoting proteasomal degradation and altering gene transcription. Activated AhR has been shown to directly associate with AR and regulate its activity in either a positive or negative manner (Ohtake et al., 2007; Swedenborg and Pongratz, 2010). Evidence further supports a functional interaction between AhR and AR; for example, Carbidopa has been reported to reduce AR protein expression through an AhR-mediated proteasomal degradation pathway, and co-immunoprecipitation experiments confirmed a physical interaction between AhR and AR (Chen et al., 2020).

At present, the mechanisms by which BaP regulates AR through AhR activation and the resulting downstream metabolic consequences remain highly controversial, representing a critical knowledge gap in this field. Existing studies report conflicting findings. Some evidence suggests that BaP may influence lipid metabolism by modulating AR activity via AhR activation (Chen et al., 2020). However, the majority of studies describe BaP as an AR antagonist. For instance, treatment of H1355 cells with 1 mmol/L BaP significantly reduced AR expression as well as testosterone-induced AR protein levels (Ghotbaddini and Powell, 2015). In contrast, Vinggaard et al. (2000) reported that low concentrations of BaP enhanced AR activity, whereas a concentration of 10 μmol/L exerted a mild inhibitory effect. These inconsistencies indicate that BaP regulation of AR is not a simple unidirectional suppression. Based on these observations, we propose a central hypothesis that BaP exerts bidirectional regulation of AR nuclear translocation and transcriptional activity through AhR activation: in the presence of androgens, BaP displays anti-androgenic effects by inhibiting AR nuclear translocation and promoting lipid accumulation; whereas under androgen-deficient conditions, BaP exhibits androgen-mimetic activity by enhancing AR nuclear translocation and fatty acid β-oxidation.

To test this hypothesis, we employed an androgen-deficient mouse model established by castration, combined with pharmacological inhibition of AhR using the selective antagonist CH223191, together with a HepG2 cell model treated with dihydrotestosterone (DHT). This integrated *in vivo* and *in vitro* approach was used to systematically elucidate the regulatory effects of the BaP–AhR–AR axis on hepatic lipogenesis pathways (SREBP2/HMGCR/LXRα) and fatty acid β-oxidation pathways (PGC-1α/CPT1A/PPARα). By clarifying how BaP modulates AR activity through AhR, this study aims to identify AhR as a key molecular switch in AR regulation and to provide novel mechanistic insights and potential therapeutic targets for the precise prevention and treatment of BaP-associated MASLD related metabolic processes.

## 2 Materials and methods

### 2.1 Experimental materials

Benzo[a]pyrene (BaP) powder (Sigma, United States; B1760), dihydrotestosterone (DHT; Solarbio, Beijing, China; ID0310), nuclear and cytoplasmic protein extraction kit (Beyotime Biotechnology, Shanghai, China; P0028), modified Oil Red O staining kit (Beyotime Biotechnology, Shanghai, China; C0158S), mouse testosterone (T) ELISA kit (No. 202407), and CH223191 (MedChemExpress; 257,156) were used in this study. Primary antibodies included AR (ab198394), CPT1A (ab220789), PPARα (ab178865), PGC-1α (ab176328), HMGCR (A19063), SREBP2 (A13049), LXRα (ab176323), Lamin B1 (ab229025), and β-actin (ab115777). All antibodies were purchased from Abcam (United Kingdom).

### 2.2 Animal model

Male KM mice were selected for this study primarily because the research aimed to investigate the effects of BaP exposure on the AhR-AR signaling axis and hepatic lipid metabolism under conditions of androgen deficiency. Male animals possess a stable androgen background, and a clear model of androgen deficiency can be established through bilateral orchiectomy; therefore, male mice were chosen to explore the underlying mechanisms. A total of 30 male KM mice, aged 6 weeks and weighing 18–22 g, were purchased from SPF Biotechnology Co., Ltd. (Beijing, China; animal production license No. SCXK (Beijing) 2024-0001). All mice were housed under specific pathogen-free (SPF) conditions at the Experimental Animal Center of Inner Mongolia Medical University (animal use license No. SYXK (Inner Mongolia) 2025-0003). Mice were randomly divided into five groups (n = 6 per group): control group, oil group (vehicle control), BaP group (0.9 mg kg^−1^ (Lou et al., 2022), gavage), BaP+gonadectomy group (bilateral orchiectomy followed by BaP 0.9 mg kg^−1^, gavage), and BaP + AhR inhibitor group (BaP 0.9 mg kg^−1^, gavage; CH223191 10 mg kg^−1^ (Li et al., 2022), intraperitoneal injection). This study employed a BaP dosage of 0.9 mg kg^−1^, selected based on the effective exposure range identified in previous animal studies—which is known to induce AhR-related signaling activation and hepatic metabolic abnormalities—while also taking into account the specific metabolic effects associated with environmental BaP exposure that are the focus of this research. The control group received no treatment, and the vehicle control group was administered an equal volume of corn oil by gavage, bilateral orchiectomy was performed prior to pharmacological treatment. Following surgery, the mice were allowed a recovery period (2 days) to ensure that endogenous testosterone levels declined and a stable castrated state was achieved before the initiation of BaP intervention. BaP was administered via oral gavage, while CH223191 was administered via intraperitoneal injection. Throughout the experiment, mice in all groups received vehicle treatments corresponding to the respective routes of administration to ensure consistency in procedural workflows and experimental conditions across the different groups. After 2 weeks of treatment, anesthesia was induced via intraperitoneal injection of 1% sodium pentobarbital (50 mg kg^−1^), and sample collection was performed. All animal experiments were approved by the Experimental Animal Management Committee and Ethics Committee of Inner Mongolia Medical University (Approval No. YKD202101183) and were conducted in accordance with animal welfare and ethical guidelines.

### 2.3 Hematoxylin–eosin (HE) and Oil Red O staining

Liver tissues from each group were fixed in 4% paraformaldehyde for 20 min. After dehydration through a graded ethanol series, tissues were subjected to HE staining.

The tissues were paraffin-embedded and sectioned into 5 μm slices. Sections were stained with hematoxylin for 5 min, differentiated with hydrochloric acid alcohol, and blued with 1% (v/v) ammonia water. After rinsing with running water, sections were stained with eosin for 30 s, dehydrated with ethanol, treated with xylene, and mounted. Images were captured using a digital pathology scanning system. Oil Red O staining was performed according to the instructions of the modified Oil Red O staining kit. Briefly, an appropriate amount of staining wash solution was added to cover the samples for 20 s and then removed. An appropriate amount of Oil Red O working solution was added, and samples were stained for 20 min. The staining solution was removed, followed by the addition of staining wash solution for 30 s. After removal of the wash solution, samples were rinsed with PBS for 20 s and observed and photographed under a microscope.

### 2.4 Enzyme-linked immunosorbent assay (ELISA)

Serum testosterone concentrations were measured using a commercial ELISA kit (Jiangsu Enzyme Immunoassay, China; Cat. No. MM-0569M2) according to the manufacturer’s instructions. Add 50 μL of mouse serum to each well of a 96-well plate, followed by 50 μL of the HRP-conjugated reagent mixture (except for blank wells). After gentle shaking, the plate was incubated at 37 °C for 1 h. Then, 200 μL washing buffer was added to each well and allowed to stand for 10 s before the liquid was completely aspirated. This washing step was repeated three times. After the final wash, residual buffer was carefully removed, and the plate was inverted and gently tapped on clean filter paper to remove remaining liquid. Subsequently, 50 μL substrate A and 50 μL substrate B were added to each well and mixed thoroughly. The plate was incubated at 37 °C for 15 min. Then, 50 μL stop solution was added, and absorbance was measured at 450 nm using a microplate reader. A standard curve was generated according to the kit instructions, and sample concentrations were calculated.

### 2.5 Plasma lipid profile

The contents of triglycerides (TG), total cholesterol (TC), low-density lipoprotein cholesterol (LDL-C), and high-density lipoprotein cholesterol (HDL-C) were measured according to the instructions of the corresponding assay kits. Absorbance was measured at 500 and 600 nm using a microplate reader. Standard curves were generated according to the kit instructions, and the concentrations of TG, TC, LDL-C, and HDL-C were calculated.

### 2.6 Measurement of aspartate aminotransferase and alanine aminotransferase (AST/ALT) activities

AST and ALT activities in mouse serum were determined according to the respective assay kit instructions. Absorbance was measured at 510 nm using a microplate reader.

Standard curves were generated based on absorbance values, and AST and ALT activities were calculated.

### 2.7 Determination of hepatic triglyceride (TG) and total cholesterol (TC) contents

Mouse liver tissues were accurately weighed and homogenized in anhydrous ethanol on ice. The homogenates were centrifuged at 4 °C and 2,500 rpm for 10 min, and the supernatants were collected for analysis. TG and TC contents were measured according to the instructions of the corresponding assay kits. Absorbance was measured at 500 nm using a microplate reader. Standard curves were generated according to the kit instructions, and TG and TC contents were calculated.

### 2.8 Cell culture and treatment

The human hepatocellular carcinoma cell line HepG2 was purchased from the Institute of Basic Medical Sciences, Chinese Academy of Medical Sciences. Cells were cultured in DMEM supplemented with 10% fetal bovine serum (FBS) and 1% penicillin–streptomycin in a humidified incubator at 37 °C with 5% CO_2_. The culture medium was replaced every 2–3 days. When cell confluence reached approximately 80%, cells were passaged using trypsin digestion. HepG2 cells in the logarithmic growth phase were seeded into 6-well plates at a density of 6 × 10^4^ cells per well and divided into four groups: (1) control group, no treatment; (2) BaP group, treated with 1 μmol L^−1^ BaP (Lou et al., 2022); (3) BaP + DHT group, treated with 1 μmol L^−1^ BaP and 1 μmol L^−1^ DHT (Ruh et al., 1975); (4) BaP + CH223191 group, treated with 1 μmol L^−1^ BaP and 1 μmol L^−1^ CH223191 (Lou et al., 2022). After 24 h of treatment, subsequent experiments were performed.

### 2.9 Quantitative real-time PCR (qPCR)

Total RNA was extracted from cells and liver tissues using TRIzol reagent. Total RNA was reverse-transcribed into cDNA according to the instructions of the reverse transcription kit, followed by qPCR analysis. The qPCR program was set as follows: (1) 95 °C for 30 s; (2) 40 cycles of 95 °C for 5 s, 60 °C for 10 s, and 72 °C for 15 s; (3) data acquisition; (4) melting curve analysis from 55 °C to 95 °C; (5) data acquisition at 20 °C for 10 s. All experiments were repeated three times. Relative mRNA expression levels of target genes were calculated using the 2^−ΔΔCt^ method, with β-actin as the internal reference. Primer sequences are listed in Table 1, human-specific primers were used for HepG2 cell experiments, whereas mouse-specific primers were applied for mouse liver tissue analysis.

**TABLE 1:** Primer sequences.

Gene	Species	Primer sequence (5′—3′)
AR	Human	Forward primer 5′-GGT​GAG​CAG​AGT​GCC​CTA​TC-3
	​	Reverse primer 5′-GAA​GAC​CTT​GCA​GCT​TCC​AC-3′
	Mouse	Forward primer 5′- TCC​AAG​ACC​TAT​CGA​GGA​GCG-3
	​	Reverse primer 5′- GTG​GGC​TTG​AGG​AGA​ACC​AT-3′
CPT1A	Human	Forward primer 5′-TCC​ACC​GTT​TGA​CTT​GTG​ACC​C-3′
	​	Reverse primer 5′-CCC​TTT​ATC​CAT​CCA​TTA​GGA​GCC​GAT-3′
	Mouse	Forward primer 5′- AGA​TCA​ATC​GGA​CCC​TAG​ACA​C-3′
	​	Reverse primer 5′- CAG​CGA​GTA​GCG​CAT​AGT​CA-3′
PPARα	Human	Forward primer 5′-ACA​GAT​GAC​GAC​CAG​GAC​AC-3′
	​	Reverse primer 5′-AAA​CGG​GAT​TGC​ATT​GTG​TGA-3′
	Mouse	Forward primer 5′- AAC​ATC​GAG​TGT​CGA​ATA​TGT​GG-3′
	​	Reverse primer 5′- CCG​AAT​AGT​TCG​CCG​AAA​GAA-3′
PGC-1α	Human	Forward primer 5′-AGG​CGG​TGC​TCG​CTT​GTG-3′
	​	Reverse primer 5′-GGG​ATA​GAT​AAA​ACA​GGA​TGC​GA-3′
	Mouse	Forward primer 5′- GGT​CCC​TAA​CCA​GGA​GTT​CG-3′
	​	Reverse primer 5′- CCA​TGA​TCC​CGT​CAA​ATT​GGG-3′
HMGCR	Human	Forward primer 5′-TGT​GGC​CAG​CAC​CAA​TAG​AG-3′
	​	Reverse primer 5′-CAA​CTG​GGC​CAC​GAG​TCA​T-3′
	Mouse	Forward primer 5′- GAG​GAC​ACG​TCC​TTG​AGG​C-3′
	​	Reverse primer 5′- TAC​ACG​GAT​GAC​CGT​GAC​C-3′
SREBP2	Human	Forward primer 5′-AGA​CTT​GGT​CAT​GGG​GAC​AG-3′
	​	Reverse primer 5′-GGG​GAG​ACA​TCA​GGG​ACA-3′
	Mouse	Forward primer 5′- CGC​TCT​GGT​TTG​TCC​GGT-3′
	​	Reverse primer 5′- GTC​AAT​CCC​GAA​AAG​AGC​GG-3′
LXRα	Human	Forward primer 5′-GAT​CGA​GGT​GAT​GCT​TCT​GGA​G-3′
	​	Reverse primer 5′-CCC​TGC​TTT​GGC​AAA​GTC​TTC-3′
	Mouse	Forward primer 5′- CAA​GGG​AGC​ACG​CTA​TGT​CTG-3′
	​	Reverse primer 5′- GGA​CAC​CGA​AGT​GGC​TTG​AG-3′
β-actin	Human	Forward primer 5′-CTA​CCT​CAT​GAA​GAT​CCT​GAC​C-3′
	​	Reverse primer 5′-CAC​AGC​TTC​TCT​TTG​ATG​TCA​C-3′
	Mouse	Forward primer 5′- CCA​GAA​GGA​CTG​TTA​TGT​GGG​A-3′
	​	Reverse primer 5′- GAC​TCC​GTG​TTC​AAT​GGG​ATA​C-3′

### 2.10 Western blot

Total protein was extracted from cells and liver tissues using RIPA lysis buffer. Nuclear and cytoplasmic proteins were extracted using the nuclear and cytoplasmic protein extraction kit. Protein samples were mixed with loading buffer at a 1:1 volume ratio and boiled for 15 min.

Proteins (20–50 μg) were separated by Tris-HCl PAGE and transferred onto PVDF membranes. Membranes were blocked in Tris-buffered saline containing 0.1% Tween-20% and 5% non-fat milk and incubated with the corresponding primary antibodies. Protein bands were detected using an Odyssey infrared imaging system. Band intensities were normalized to β-actin or Lamin B1 and quantified using ImageJ software.

### 2.11 Statistical analysis

Data are expressed as mean ± standard deviation (SD). ImageJ was used for image visualization and analysis, and GraphPad Prism 7 was used for data analysis. The normality of the data was assessed using the Shapiro–Wilk test, and homogeneity of variance was evaluated before parametric analyses. For data that satisfied the assumptions of normality and homogeneity of variance, comparisons between two groups were performed using an unpaired two-tailed Student’s t-test. For comparisons among multiple groups, one-way analysis of variance (ANOVA) followed by Tukey’s multiple comparisons test was applied. A value of *P* < 0.05 was considered statistically significant.

## 3 Results

### 3.1 Effects of BaP exposure on hepatic lipid metabolism in mice under different androgen status conditions

Successful establishment of the model was confirmed by a significant reduction in serum testosterone levels in gonadectomized mice compared to the oil-treated control group (Figure 1A). No significant difference was observed between the vehicle control group and Control group. Histological evaluation of liver tissues by hematoxylin–eosin (HE) staining revealed no obvious morphological alterations in hepatocytes from the oil-treated group. In contrast, BaP exposure induced marked hepatocellular injury, characterized by disordered cell arrangement and pronounced vacuolar degeneration. Notably, mice treated with BaP after gonadectomy exhibited attenuated hepatic lesions compared with the BaP group, with reduced vacuolar degeneration. Furthermore, Oil Red O staining demonstrated that BaP exposure significantly increased hepatic lipid accumulation, as indicated by a higher percentage of lipid droplet area compared with the control group. In contrast, gonadectomized mice exposed to BaP showed a significant reduction in hepatic lipid droplet area compared with the BaP group (Figures 1B,C).

**FIGURE 1:**
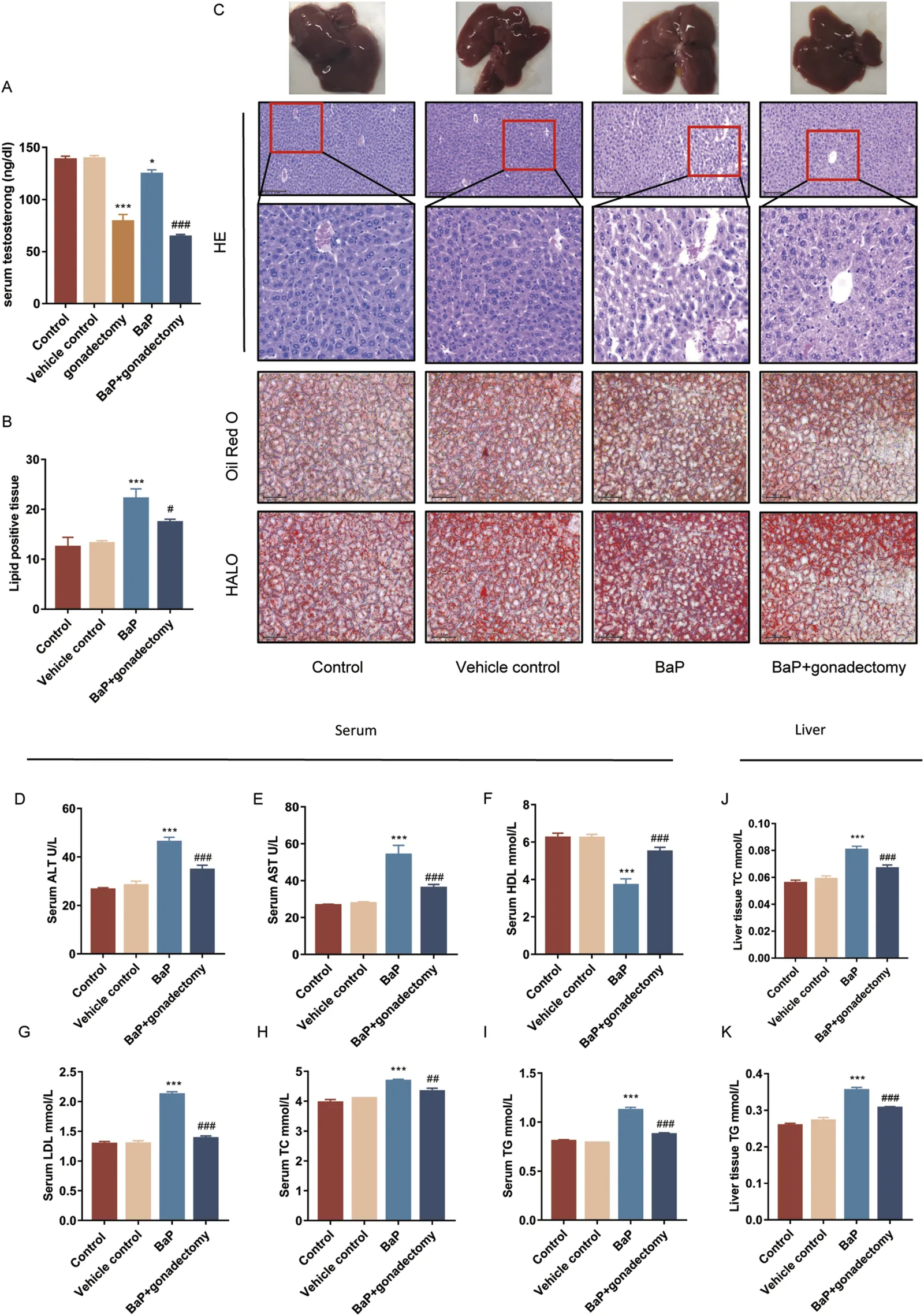
Effects of BaP exposure on hepatic lipid metabolism in mice under different androgen status conditions. **(A)** Serum testosterone concentrations; **(B)** Quantification of Oil Red O-positive lipid droplet area in liver sections using HALO software; **(C)** Representative hematoxylin and eosin (H and E) staining, Oil Red O staining, and quantitative analysis of hepatic lipid droplet area; **(D)** Serum alanine aminotransferase (ALT) levels; **(E)** Serum aspartate aminotransferase (AST) levels; **(F)** Serum high-density lipoprotein cholesterol (HDL-C) levels; **(G)** Serum low-density lipoprotein cholesterol (LDL-C) levels; **(H)** Serum total cholesterol (TC) levels; **(I)** Serum triglyceride (TG) levels; **(J)** Hepatic total cholesterol (TC) levels; **(K)** Hepatic triglyceride (TG) levels. Data are presented as mean ± SD (n = 6 per group). Scale bars = 100 μm. Compared with the vehicle control group, *P < 0.05, ***P < 0.001; compared with the BaP group, ^*#*^ *P <* 0.05*,* ^*##*^ *P <* 0.01*,* ^*###*^ *P <* 0.001.

The results of serum biochemical analyses showed that, compared with the vehicle control group, BaP exposure significantly increased serum ALT and AST activities. In contrast, ALT and AST levels were significantly reduced in the BaP + gonadectomy group compared with the BaP group (Figures 1D,E). In addition, measurements of serum TG, TC, LDL-C, and HDL-C levels showed that all lipid parameters in the BaP + gonadectomy group were lower than those in the BaP group. Further determination of hepatic TC and TG contents yielded results consistent with those observed in serum (Figures 1F–K). These findings suggest that BaP exposure promotes hepatic injury, lipid accumulation, and dyslipidemia in the presence of androgens. However, androgen deficiency attenuates BaP-induced hepatic lipid metabolic disturbances, indicating that androgen status plays an important role in regulating the metabolic effects of BaP.

### 3.2 Effects of BaP exposure on AR subcellular distribution and hepatic lipid metabolism-related gene expression in intact and castrated mice

To investigate whether BaP affects AR nuclear translocation by interfering with androgen–AR binding, We analyzed AR protein expression in nuclear and cytoplasmic fractions of mouse liver tissues by Western blotting (Figure 2A). BaP exposure significantly reduced both nuclear and cytoplasmic AR protein levels compared to the vehicle control group. Compared with the BaP group, nuclear AR protein expression was significantly increased in mice treated with the BaP + gonadectomy group (Figures 2A–C). These results indicate that in the presence of androgens, BaP treatment affects the distribution of AR between the nucleus and the cytoplasm, suggesting that BaP may be involved in regulating the nuclear localization of AR and exhibiting anti-androgenic effects. In contrast, under androgen-deficient conditions, BaP promotes AR nuclear translocation. Further analysis of lipid metabolism-related gene expression revealed that, compared with the BaP group, the BaP + gonadectomy group showed significantly increased mRNA levels of β-oxidation-related genes, including PGC-1α, CPT1A, and PPARα. Consistently, the protein expression levels of these β-oxidation-related factors were also elevated. In contrast, the mRNA and protein expression levels of lipid synthesis-related factors, including SREBP2, HMGCR, and LXRα, were significantly decreased in the BaP + gonadectomy group (Figures 2D–H). These findings were consistent with the changes observed in AR nuclear translocation. Under androgen-present conditions, reduced AR nuclear translocation was accompanied by decreased β-oxidation and enhanced lipid synthesis. Conversely, under androgen-deficient conditions, increased AR nuclear translocation was associated with enhanced β-oxidation and suppressed lipid synthesis. Collectively, these results suggest that BaP regulates AR nuclear translocation in an androgen-dependent manner, thereby modulating downstream lipid metabolism-related pathways and influencing hepatic lipid accumulation.

**FIGURE 2:**
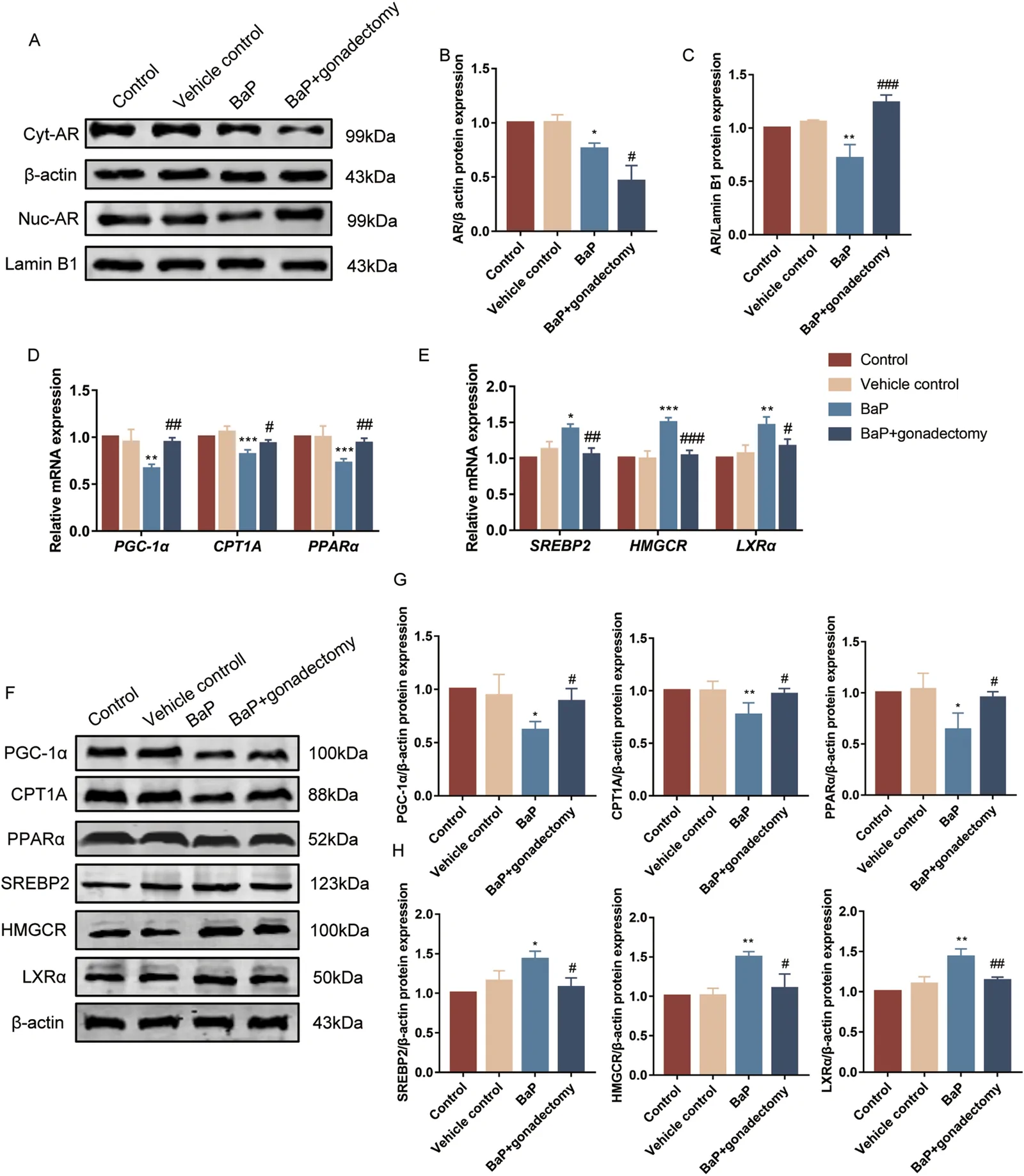
Effects of BaP exposure on AR subcellular distribution and hepatic lipid metabolism-related gene expression in intact and castrated mice. **(A)** Representative Western blot bands showing nuclear and cytoplasmic AR protein expression in liver tissues; **(B,C)** Quantification of nuclear and cytoplasmic AR protein levels; **(D)** Relative mRNA expression levels of β-oxidation-related genes (PGC-1α, CPT1A, and PPARα) in liver tissues; **(E)** Relative mRNA expression levels of lipid synthesis-related genes (SREBP2, HMGCR, and LXRα); **(F)** Representative Western blot bands of lipid synthesis- and β-oxidation-related proteins in liver tissues; **(G)** Quantification of β-oxidation-related protein expression; **(H)** Quantification of lipid synthesis-related protein expression. Data are presented as mean ± SD (n = 6 per group). Compared with the vehicle control group, *P < 0.05, **P < 0.01, ***P < 0.001; compared with the BaP group, ^#^ *P* < 0.05, ^##^ *P* < 0.01, ^###^ *P* < 0.001.

### 3.3 Effects of AhR inhibition on BaP-induced alterations in hepatic lipid metabolism and AR-related responses in mice

HE staining results showed that hepatocellular injury was evident in the BaP group, with extensive vacuole formation, which may be attributed to BaP-induced lipid metabolism disorder. Compared with the BaP group, pathological liver injury was alleviated and hepatocellular structure tended to normalize after AhR inhibition. Oil Red O staining showed that, compared with the vehicle control group, the percentage of lipid droplet area was significantly increased in the BaP group. In contrast, the BaP + CH223191 group exhibited a significant reduction in lipid droplet area compared with the BaP group (Figures 3A,B). Biochemical assay results further showed that inhibition of AhR blocked BaP-induced dyslipidemia and liver injury in mice (Figures 3C–J). These results demonstrate that AhR activation is critically involved in BaP-induced hepatic steatosis and liver injury, and that pharmacological targeting of AhR effectively mitigates these adverse effects.

**FIGURE 3:**
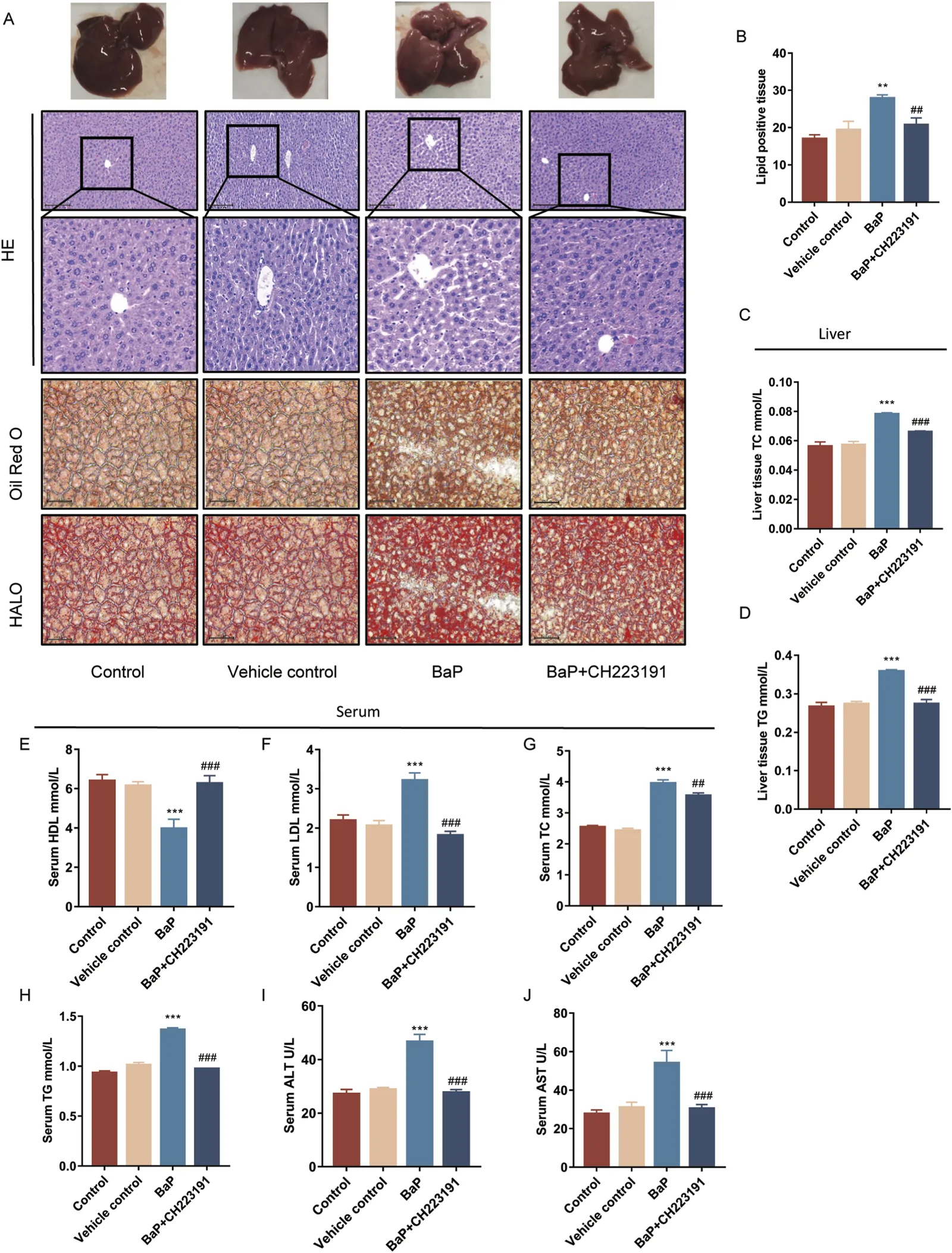
Effects of AhR inhibition on BaP-induced alterations in hepatic lipid metabolism and AR-related responses in mice. **(A)** Representative hematoxylin and eosin (H and E) staining and Oil Red O staining of liver tissues; **(B)** Quantitative analysis of Oil Red O-positive lipid droplet area; **(C)** Hepatic total cholesterol (TC) levels; **(D)** Hepatic triglyceride (TG) levels; **(E)** Serum high-density lipoprotein cholesterol (HDL-C) levels; **(F)** Serum low-density lipoprotein cholesterol (LDL-C) levels; **(G)** Serum total cholesterol (TC) levels; **(H)** Serum triglyceride (TG) levels; **(I)** Serum aspartate aminotransferase (AST) levels; **(J)** Serum alanine aminotransferase (ALT) levels. Data are presented as mean ± SD (n = 6 per group). Scale bars = 100 μm. Compared with the vehicle control group, **P < 0.01, ***P < 0.001; compared with the BaP group, ^*##*^ *P <* 0.01*,* ^*###*^ *P <* 0.001.

### 3.4 Effects of AhR inhibition on AR expression and hepatic lipid metabolism-related responses following BaP exposure in mice

To further establish the role of AhR in mediating BaP’s effects on AR signaling, we examined AR protein expression following pharmacological inhibition of AhR with CH223191. Compared with the vehicle control group, AR protein expression was decreased in the BaP group. However, inhibition of AhR by CH223191 significantly increased AR protein expression compared with the BaP group. These results indicate that in the presence of androgens, BaP exerts anti-androgenic effects by inhibiting AR expression, whereas inhibition of AhR blocks the effects of BaP and restores AR expression (Figures 4A,B). Compared with the BaP group, the mRNA expression of PGC-1α, CPT1A, and PPARα was increased in the BaP + CH223191 group, and the protein expression levels were generally consistent with the mRNA expression patterns. In contrast, compared with the BaP group, the mRNA expression of SREBP2, HMGCR, and LXRα was decreased in the BaP + CH223191 group, and protein expression levels showed similar trends (Figures 4C–G). Following AhR inhibition, the inhibitory effect of BaP on androgen signaling was attenuated, as evidenced by increased AR expression, upregulation of β-oxidation-related genes, and downregulation of cholesterol synthesis-related genes. These results demonstrate that BaP regulates AR expression through AhR activation, thereby modulating the expression of downstream genes governing hepatic lipid metabolism. Pharmacological inhibition of AhR effectively abrogates these effects, restoring AR expression.

**FIGURE 4:**
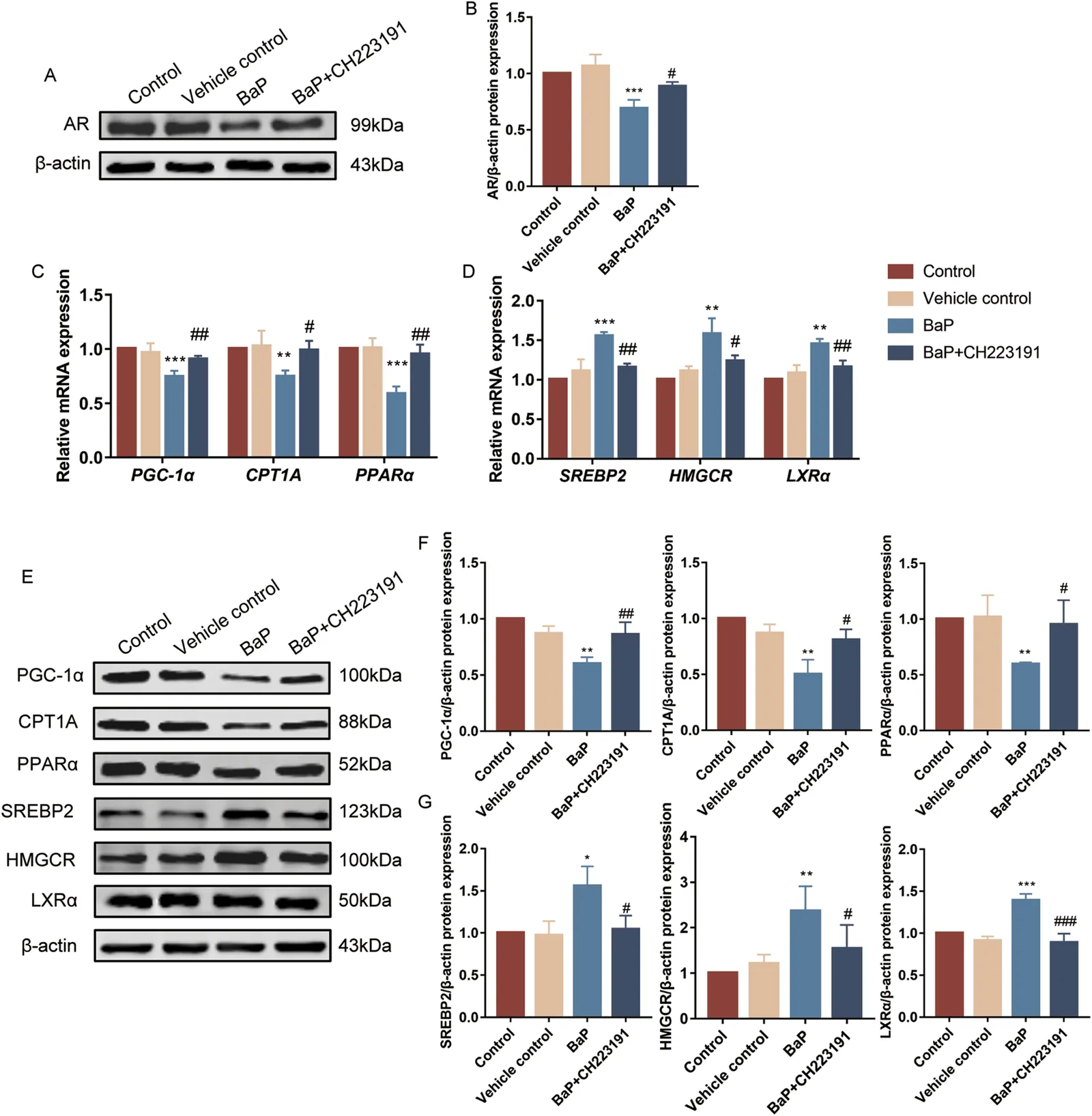
Effects of AhR inhibition on AR expression and hepatic lipid metabolism-related responses following BaP exposure in mice. **(A)** Representative Western blot bands showing AR protein expression in liver tissues; **(B)** Quantification of AR protein expression; **(C)** Relative mRNA expression levels of β-oxidation-related genes (PGC-1α, CPT1A, and PPARα); **(D)** Relative mRNA expression levels of lipid synthesis-related genes (SREBP2, HMGCR, and LXRα); **(E)** Representative Western blot bands of lipid synthesis- and β-oxidation-related proteins in liver tissues; **(F)** Quantification of β-oxidation-related protein expression; **(G)** Quantification of lipid synthesis-related protein expression. Data are presented as mean ± SD (n = 6 per group).Compared with the vehicle control group, *P < 0.05, **P < 0.01, ***P < 0.001; compared with the BaP group, ^#^ *P* < 0.05, ^##^ *P* < 0.01, ^###^ *P* < 0.001.

### 3.5 Effects of BaP exposure on AR subcellular distribution and lipid metabolism-related responses in HepG2 cells with or without DHT treatment

To determine whether androgen availability influences the effects of BaP on AR signaling and lipid metabolism, HepG2 cells were treated with BaP under DHT-free conditions or androgen-supplemented (1 μmol L^−1^ DHT) conditions, followed by analysis of AR protein expression in nuclear and cytoplasmic fractions. (Figures 5A–C). Under DHT-free conditions, BaP increased cytoplasmic AR expression and promoted AR nuclear translocation, as indicated by elevated AR protein levels in both cytoplasmic and nuclear fractions, suggesting an androgen-related regulatory effect of BaP. These findings suggest that, in the absence of androgens, BaP may promote AR nuclear translocation—potentially through AhR-mediated pathways—while also influencing AR protein stability or synthesis. Under DHT-supplemented conditions, the BaP + DHT group exhibited decreased nuclear AR protein levels and increased cytoplasmic AR protein levels compared with the BaP group (without DHT treatment). These results indicate that the presence of DHT alters the effect of BaP on AR localization, leading to reduced AR nuclear translocation. Consistent with the alterations in AR nuclear translocation, the expression of downstream lipid metabolism–related genes also showed androgen-dependent bidirectional regulation (Figures 5D–H). Compared with the BaP group, the BaP + DHT group exhibited decreased expression of β-oxidation-related genes and proteins (CPT1A, PPARα, and PGC-1α) and increased expression of lipid synthesis-related genes and proteins (SREBP2, HMGCR, and LXRα). Collectively, these cellular data corroborate the *in vivo* findings, demonstrating that BaP exerts context-dependent bidirectional regulation of AR signaling: under DHT-free conditions, BaP promotes AR nuclear translocation and enhances fatty acid oxidation, exhibiting androgen-related regulatory effect; conversely, in the presence of androgens, BaP inhibits AR nuclear translocation and promotes lipid synthesis, exerting anti-androgenic effects.

**FIGURE 5:**
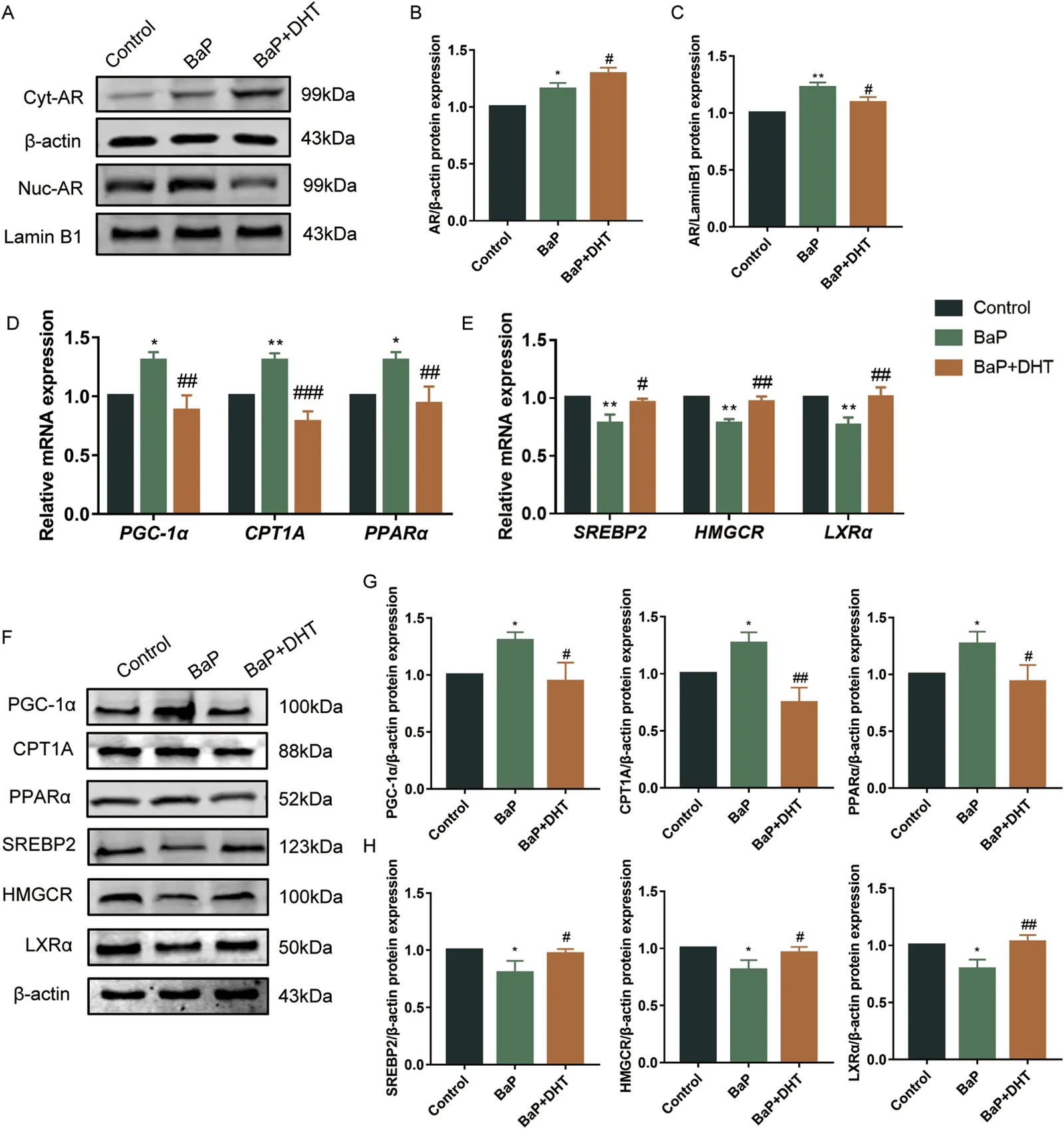
Effects of BaP exposure on AR subcellular distribution and lipid metabolism-related responses in HepG2 cells with or without DHT treatment. **(A)** Representative Western blot bands showing nuclear and cytoplasmic AR protein expression in HepG2 cells; **(B,C)** Quantification of nuclear and cytoplasmic AR protein levels; **(D)** Relative mRNA expression levels of β-oxidation-related genes (PGC-1α, CPT1A, and PPARα) in HepG2 cells; **(E)** Relative mRNA expression levels of lipid synthesis-related genes (SREBP2, HMGCR, and LXRα) in HepG2 cells; **(F)** Representative Western blot bands of lipid synthesis- and β-oxidation-related proteins in HepG2 cells; **(G)** Quantification of β-oxidation-related protein expression; **(H)** Quantification of lipid synthesis-related protein expression. Data are presented as mean ± SD (n = 6 independent biological replicates). Compared with the control group, *P < 0.05, **P < 0.01; compared with the BaP group, ^#^ *P* < 0.05, ^##^ *P* < 0.01, ^###^ *P* < 0.001.

### 3.6 Effects of AhR inhibition on AR-related responses and lipid metabolism-associated molecules following BaP exposure in HepG2 cells

To further investigate the role of AhR in mediating the effects of BaP on AR signaling, HepG2 cells were pretreated with the selective AhR antagonist CH223191 before BaP exposure. Western blot analysis showed that BaP treatment significantly increased AR protein expression compared with the Control group. Notably, CH223191 pretreatment attenuated this effect, as indicated by reduced AR expression in the BaP + CH223191 group compared with the BaP group (Figures 6A,B). These findings suggest that BaP modulates AR expression in HepG2 cells through an AhR-dependent mechanism.

**FIGURE 6:**
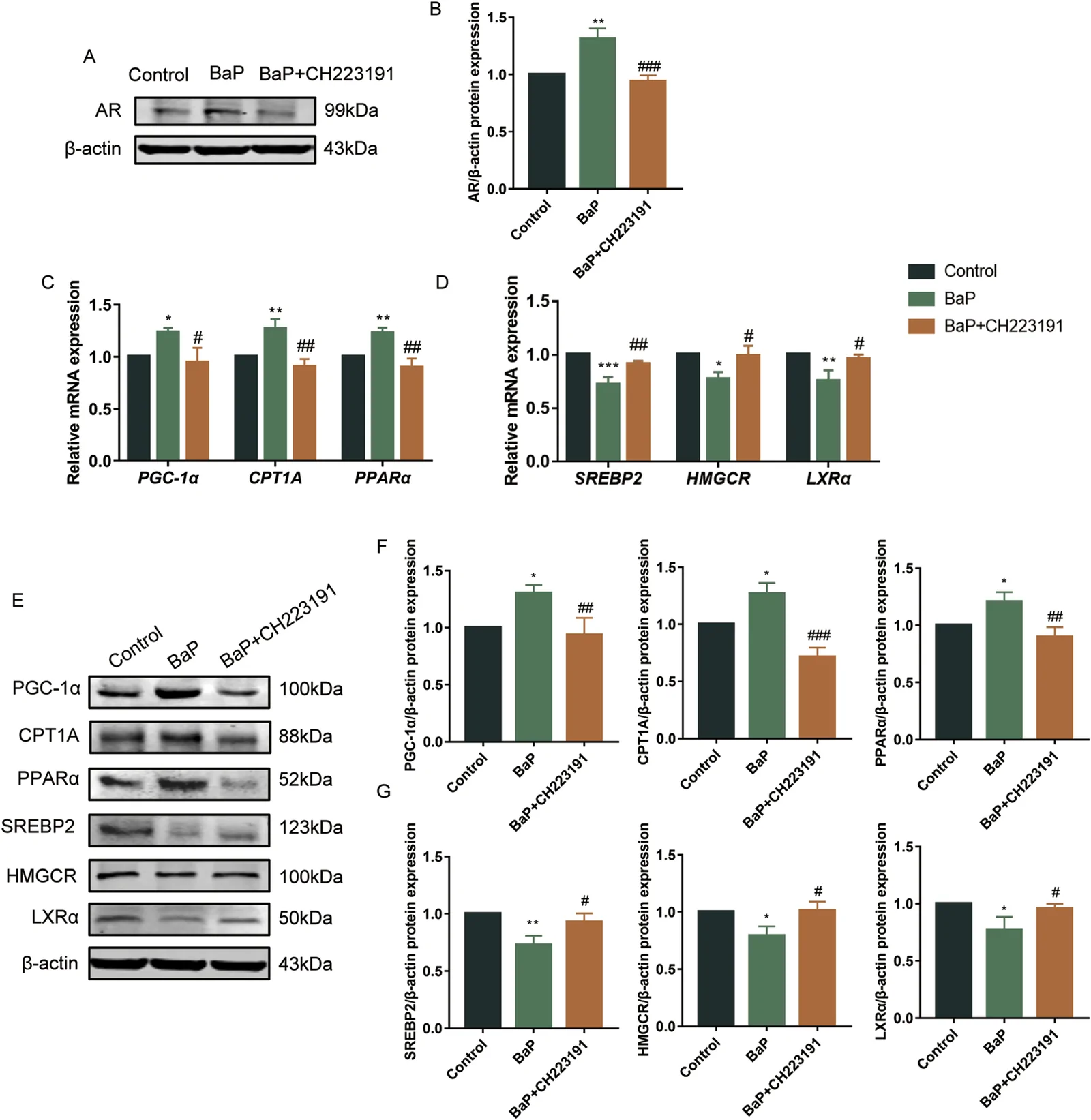
Effects of AhR inhibition on AR-related responses and lipid metabolism-associated molecules following BaP exposure in HepG2 cells. **(A)** Representative Western blot bands showing AR protein expression in HepG2 cells; **(B)** Quantification of AR protein expression; **(C)** Relative mRNA expression levels of β-oxidation-related genes (PGC-1α, CPT1A, and PPARα) in HepG2 cells; **(D)** Relative mRNA expression levels of lipid synthesis-related genes (SREBP2, HMGCR, and LXRα) in HepG2 cells; **(E)** Representative Western blot bands of lipid synthesis- and β-oxidation-related proteins in HepG2 cells; **(F)** Quantification of β-oxidation-related protein expression; **(G)** Quantification of lipid synthesis-related protein expression. Data are presented as mean ± SD (n = 6 independent biological replicates). Compared with the control group, *P < 0.05, **P < 0.01, ***P < 0.001; compared with the BaP group, ^#^ *P* < 0.05, ^##^ *P* < 0.01, ^###^ *P* < 0.001.

Consistent with the changes in AR expression, qPCR and Western blot analyses showed that CH223191 pretreatment attenuated BaP-induced alterations in lipid metabolism-related factors. Compared to the BaP group, the BaP + CH223191 group exhibited decreased mRNA and protein expression of β-oxidation-related genes (PGC-1α, CPT1A, PPARα), whereas the expression of lipid synthesis-related genes (SREBP2, HMGCR, LXRα) was increased (Figures 6C–G).

These cellular data corroborate the *in vivo* findings presented in Figure 4, demonstrating that BaP modulates AR expression through AhR activation, thereby regulating the expression of downstream genes governing hepatic lipid metabolism in an AhR-dependent manner.

## 4 Discussion

Driven by high-fat diets, unhealthy lifestyles, and environmental pollution, the incidence of hepatic lipid metabolic diseases has been increasing year by year and has become a global public health challenge (Pipitone et al., 2023). Benzo[a]pyrene (BaP), a typical representative of polycyclic aromatic hydrocarbons (PAHs), is currently recognized as one of the most potent carcinogenic markers. Recent studies have demonstrated that, in addition to its carcinogenic effects, BaP can significantly increase hepatic lipid accumulation, insulin resistance, and the risk of MASLD by disrupting metabolic homeostasis (Holme et al., 2023; Šimečková et al., 2022; Bukowska et al., 2022). Consistent with these reports, the present study observed significant hepatotoxicity following BaP exposure in both *in vivo* and *in vitro* models. BaP is a classical exogenous ligand of the aryl hydrocarbon receptor (AhR) (Diao et al., 2025). Consistent with our previous systematic studies, BaP exposure effectively activates AhR signaling and significantly upregulates the expression of the classic downstream target gene CYP1A1. The AhR-inducing effect of BaP and the inhibitory effect of the AhR antagonist CH223191 on AhR activation and CYP1A1 expression have been fully validated in our published work (Lou et al., 2022; Zhang et al., 2022), which solidly confirms the successful activation of AhR in the present BaP-exposed experimental system. Based on existing literature, ligand-activated AhR can form a ubiquitin ligase complex with components such as CUL4B and participate in regulating the stability of certain target proteins (Ohtake et al., 2009). Therefore, we hypothesize that AhR activation might influence AR protein levels through a similar mechanism. In this study, inhibition of AhR by CH223191 under BaP exposure conditions was able to regulate AR expression, thereby affecting hepatic lipid metabolism and the expression of related genes. Therefore, AhR may serve as a core molecular switch through which BaP regulates AR function. However, as this study did not directly examine the AhR-AR interaction, AR ubiquitination, or proteasome-dependent degradation, this hypothesis requires further experimental validation.

The interaction between AhR and steroid hormone receptors plays a central role in the regulation of multiple physiological processes (Bustani et al., 2025). AR is a member of the steroid hormone nuclear receptor family, and androgens and AR play important roles in the regulation of glucose and lipid metabolism (Yin et al., 2023). AR shares similar structural domains with other nuclear receptors, which largely mediate the binding of androgens to androgen response elements (AREs) and thereby regulate various physiological functions (Bartsch et al., 2021). In the absence of androgen binding, AR exists in the cytoplasm as a complex with molecular chaperones such as HSP40, HSP70, and HSP90. Upon androgen binding, conformational changes occur in the AR protein, leading to dimerization and translocation into the nucleus. Nuclear translocation enables AR to function as a transcription factor by binding to AREs in the regulatory regions of target genes. Binding of testosterone to AR promotes conformational changes in the androgen–AR complex, facilitating its association with AREs, resulting in the following effects: (1) reduction of hepatic cholesterol synthesis, mainly by increasing the phosphorylation level of HMGCR, thereby promoting HMGCR degradation and reducing LXRα- and SREBP2-mediated cholesterol synthesis; (2) enhancement of hepatic lipid consumption, primarily through upregulation of PGC-1α, CPT1A, and PPARα expression, thereby strengthening fatty acid β-oxidation and reducing hepatic lipid accumulation; and (3) *in vivo*, testosterone is converted into dihydrotestosterone by 5α-reductase and then binds to AR in target cells to exert its effects, while deficiency of 5α-reductase can lead to hepatic steatosis in male mice (Zhang et al., 2013). These studies indicate that activation of AR exerts protective effects on hepatic lipid metabolism.

In addition, studies have reported that the environmental hazard factor TCDD exhibits androgen-mimetic activity, such as inducing the expression of AR-regulated genes in the absence of androgens (Sanada et al., 2020). The present study showed that under androgen-deficient conditions (Rowe et al., 2023), BaP also has androgen-related regulatory effects, promoting AR expression in the nucleus, thereby upregulating the expression of PGC-1α, CPT1A, and PPARα, downregulating the expression of SREBP2, HMGCR, and LXRα, and ultimately reducing hepatic lipid content. However, in the presence of androgens, BaP exerted anti-androgenic effects, inhibited AR nuclear expression, and increased hepatic lipid content.

There remains some controversy regarding the impact of BaP on AR signaling. Some studies report that BaP exerts anti-androgenic effects, whereas others suggest it exhibits concentration-dependent, bidirectional regulatory effects. The results of this study indicate that the influence of BaP on AR-related pathways likely depends on various factors, including endogenous androgen levels, AhR activation status, exposure dosage, and differences in experimental models. In particular, under conditions of androgen deprivation, the AR signaling environment is altered, potentially leading to BaP-induced receptor regulation patterns that differ from those observed under normal androgenic conditions. Consequently, the androgen-like or anti-androgenic effects of BaP observed in previous studies may represent specific responses tailored to distinct biological contexts, rather than mutually exclusive modes of action.

This study provides a new perspective for understanding the effects of environmental pollutants on metabolic diseases. However, several limitations should be acknowledged. For instance, this study did not employ dextran-coated charcoal to strip serum and establish stringent androgen-depleted culture conditions, the *in vitro* results primarily support the role of BaP in modulating AR signaling and related metabolic pathways under varying levels of DHT stimulation, rather than fully replicating the *in vivo* castration model. Furthermore, since this study did not utilize radioligand competitive binding assays, surface plasmon resonance (SPR) analysis, or ARE reporter gene assays, further research is required to verify whether BaP directly interacts with the AR ligand-binding domain and whether it affects AR transcriptional activity. As a group treated with castration alone (plus vehicle) was not included, we could not fully distinguish between metabolic alterations caused by androgen deprivation itself and effects specifically associated with BaP exposure; therefore, future studies should incorporate a castration control group and androgen supplementation experiments to further elucidate the specific role of androgen signaling in BaP-induced metabolic remodeling. Since the study utilized only male mice, the findings cannot be directly extrapolated to females. In female animals, estrogen receptor signaling and androgen receptor (AR) signaling jointly regulate hepatic metabolism, and BaP activation of the aryl hydrocarbon receptor (AhR) may further influence estrogen-related pathways. Consequently, future research should employ female and ovariectomized animal models to compare differences in BaP-induced metabolic alterations and endocrine regulatory mechanisms across sexes. The present study focused primarily on the effects of BaP exposure on lipid metabolism disorder and their associated molecular characteristics, without investigating the subsequent pathological progression to more advanced liver diseases such as steatohepatitis, cirrhosis, or hepatocellular carcinoma. Future research should address these gaps by the intrinsic relationship between this pathway and the occurrence and progression of liver diseases, aiming to systematically elucidate the development and progression of metabolic diseases mediated by environmental pollutant exposure.

In conclusion, this study demonstrates that BaP exerts microenvironment-dependent bidirectional regulation of AR nuclear translocation and transcriptional activity through AhR activation. Under physiological androgen levels, the BaP–AhR axis exhibits anti-androgenic effects by inhibiting AR nuclear translocation and downregulating its transcriptional activity, leading to upregulation of lipid synthesis–related genes (SREBP2, HMGCR, and LXRα) and downregulation of β-oxidation–related genes (PGC-1α, CPT1A, and PPARα), ultimately inducing hepatic lipid accumulation and metabolic disorders. In contrast, under androgen-deficient conditions, BaP exhibits androgen-related regulatory effect by promoting AR nuclear translocation and enhancing its transcriptional function, resulting in downregulation of lipid synthesis–related genes (SREBP2, HMGCR, and LXRα) and upregulation of β-oxidation–related genes (PGC-1α, CPT1A, and PPARα), thereby alleviating hepatic steatosis (Figure 7). This finding reveals AhR as a core molecular switch regulating AR function and provides a new theoretical framework for understanding the interaction between environmental pollutants and sex hormones in the pathogenesis of lipid metabolism disorder, as well as for the development of targeted prevention and intervention strategies.

**FIGURE 7:**
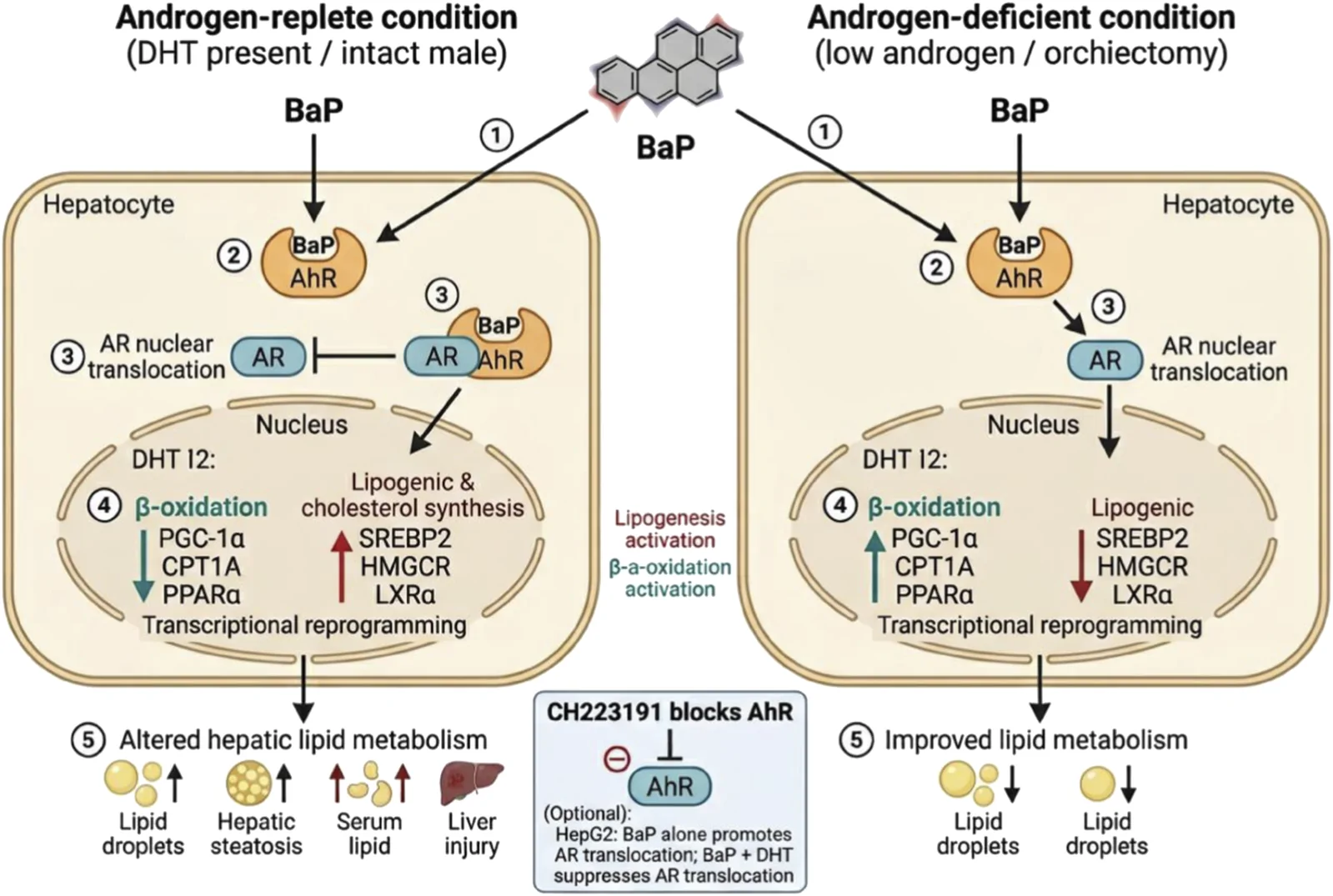
Graphical illustration of BaP-induced alterations in hepatic lipid metabolism under different androgen status conditions and the potential involvement of AhR-AR-related signaling.

## Data Availability

The original contributions presented in the study are included in the article/supplementary material, further inquiries can be directed to the corresponding authors.
